# Research Advances and Prospects of Orphan Genes in Plants

**DOI:** 10.3389/fpls.2022.947129

**Published:** 2022-07-08

**Authors:** Mingliang Jiang, Xiaonan Li, Xiangshu Dong, Ye Zu, Zongxiang Zhan, Zhongyun Piao, Hong Lang

**Affiliations:** ^1^School of Agriculture, Jilin Agricultural Science and Technology College, Jilin, China; ^2^College of Horticulture, Shenyang Agricultural University, Shenyang, China; ^3^School of Agriculture, Yunnan University, Kunming, China

**Keywords:** plant, orphan genes, identification, functions, research advances, prospects

## Abstract

Orphan genes (*OGs*) are defined as genes having no sequence similarity with genes present in other lineages. *OGs* have been regarded to play a key role in the development of lineage-specific adaptations and can also serve as a constant source of evolutionary novelty. These genes have often been found related to various stress responses, species-specific traits, special expression regulation, and also participate in primary substance metabolism. The advancement in sequencing tools and genome analysis methods has made the identification and characterization of *OGs* comparatively easier. In the study of *OG* functions in plants, significant progress has been made. We review recent advances in the fast evolving characteristics, expression modulation, and functional analysis of *OGs* with a focus on their role in plant biology. We also emphasize current challenges, adoptable strategies and discuss possible future directions of functional study of *OGs*.

## Introduction

Orphan genes (*OGs*) also known as taxonomically restricted genes (*TRGs*), more commonly referred to as ORFan genes are the sequences with no significant sequence’s similarity to any other ORF in a specified database. These are the genes that display no substantial sequence similarity outside their species and are unique to a particular taxonomic group (Siew and Fischer, 2003; Yin and Fischer, 2008; Ekstrom and Yin, 2016; Chen et al., 2020; Vakirlis et al., 2020; Weisman et al., 2020). Most *OGs* may evolve quickly enough that their similarity to other genes is lost after a certain evolutionary distance (Cai et al., 2006). The development of such genes may occur as a result of rearrangement and duplication processes followed by a rapid divergence event. However, the *de novo* model of evolution from non-coding genomic areas has also been considered as one of many possible mechanisms, appears more common than previously thought, and *de novo* gene birth provides an important mechanism for functional evolution (Khalturin et al., 2009; Toll-Riera et al., 2009; Tautz and Domazet-Lošo, 2011; Carvunis et al., 2012; Mahalak and Chamberlin, 2015; Schlötterer, 2015). Phylogenetically, the *OGs* have been found associated with lineage-specific characteristics, adaptations, and biological functions, and *OGs* have been reported to be involved in stress response, development of species-specific traits and primary substance metabolism (Wissler et al., 2013; Arendsee et al., 2014; Mukherjee et al., 2015; Jiang et al., 2018).

With the release of numerous plant genomes and transcriptomes data makes it easy to identify the *OGs* in a particular taxon. The BLAST (Basic Local Alignment Search Tool) algorithm is widely used in the screening and identification of *OGs* (Graham et al., 2004; Prabh and Rödelsperger, 2019). Studies have revealed that every species have been known to possess a considerable fraction of *OGs* (up to 30%) of the gene catalog in all sequenced genomes (Arendsee et al., 2014; Palmieri et al., 2014). It has been noted that the formation of *OGs* mostly occurs due to poorly assembled genomes that have been partially annotated by gene predictions (Prabh and Rödelsperger, 2016). Hence, the assembled genome’s quality and integrity are crucial for precise *OG* identification. Several studies have been conducted for the identification and characterization of the *OGs* in plants (including plants such as *Brassica rapa*, *Arabidopsis thaliana*, *Oryza sativa*, *Populus trichocarpa*, *Vigna unguiculata*, and *Citrus sinensis*) (Campbell et al., 2007; Yang et al., 2009; Lin et al., 2010; Donoghue et al., 2011; Xu et al., 2015; Jiang et al., 2018). Nonetheless, as compared to evolutionarily conserved genes, *OGs* are shorter, evolve faster, and have lower and more tissue-specific expression (Cai and Petrov, 2010; Palmieri et al., 2014; Li T. P. et al., 2021). In the absence of functional motifs, identifiable folds, and recognizable domains, the functional characterization of *OGs* becomes extremely mysterious.

This review mainly focuses on four segments, the first segment includes the identification and fast evolving characteristics of *OGs* in plants. The second segment covers the special expression regulatory mode of *OGs*, and the third segment covers the mysterious functions of *OGs*. Moreover, the study also entails a detailed description of current challenges, adoptable strategies and possible future directions of functional study of *OGs*.

## Orphan Genes Identification and Its Fast Evolving Characteristics

### Identification of Orphan Genes in Plants

Recent technological advancements have resulted in tremendous growth in genomics, with a vast number of genes being annotated. The rapid accumulation of genomic data on a large variety of plants will allow increasing integrity of identification of *OGs*. So far the presence of *OGs* has been reported in many plants or lineages [such as *B. rapa* (Jiang et al., 2018), *A. thaliana* (Yang et al., 2009; Lin et al., 2010; Donoghue et al., 2011; Cui et al., 2015), *O. sativa* (Guo et al., 2007; Yang et al., 2009; Cui et al., 2015; Jin et al., 2019), *P. trichocarpa* (Yang et al., 2009), *V. unguiculata* (Li G. et al., 2019), Poaceae (Campbell et al., 2007), *Aegiceras corniculatum* (Ma et al., 2021), *C. sinensis* (Xu et al., 2015), *Amaranthus hypochondriacus* (Cabrales-Orona and Délano-Frier, 2021), *Camellia sinensis* (Zhao and Ma, 2021), and eight Cucurbitaceae family members (Ma et al., 2022)]. Every sequenced genome contains *OGs* whose origins are obscure because of the absence of homologs (Blanco-Melo et al., 2016).

In various lineages or species, the percentage of genes that are *OGs* varies greatly, making up less than 1 to 17 percent of all genes in a genome, with 1–5% being typically normal (Table 1). The discrepancy in such a case can be attributed to the ongoing sequencing of multiple genomes, as well as the variable evolutionary distance among the species taken into consideration and their nearest sequenced relatives (Arendsee et al., 2014; Jiang M. et al., 2020). Similarly, multiple versions of the reference genome may lead to a distinct number of *OGs*, as in the case of the identification of *Arabidopsis* (Yang et al., 2009; Donoghue et al., 2011), and *Populus* (Yang et al., 2009; Lin et al., 2013).

**TABLE 1:** Identification of orphan genes (*OGs*) in plants.

Lineages or species	Algorithms	*E*-value cutoff	Number of *OGs*	Percentage	References
*Brassica rapa*	BLAST	1E-03	529 real A subgenome-specific *BSGs* (*Brassica*-specific genes) (these 529 *BSGs* also named as *BrOGs*)	1%	Jiang et al., 2018; Jiang M. et al., 2020
*Arabidopsis thaliana*	BLAST	1E-03	958 lineage-specific genes (*LSGs*)	3%	Donoghue et al., 2011
	BLAST	1E-01	165 *Arabidopsis*-specific genes	1%	Yang et al., 2009
	BLAST	1E-05	1,324 *Arabidopsis* lineage-specific genes (*ALSGs*)	5%	Lin et al., 2010
	BLAST	1E-03	861 species-specific orphan genes (*SSOG*)	3.14%	Cui et al., 2015
*Oryza sativa*	BLAST	1E-01	638 *Oryza*-specific genes	1%	Yang et al., 2009
	BLAST	1E-04	1,926 *OGs*	3%	Guo et al., 2007
	BLAST and BLAT	1E-02	37 *OGs*	0.0006%	Jin et al., 2019
	BLAST	1E-03	478 *SSOG*	1.18%	Cui et al., 2015
*Populus trichocarpa*	BLAST	1E-01	109 *Populus*-specific genes	0.2%	Yang et al., 2009
	BLAST	1E-02	40 *Populus trichocarpa*-specific genes (*PtSS*)	0.3%	Lin et al., 2013
*Vigna unguiculata*	BLAST and Microarray-based genome hybridization	1E-10	578 cowpea *OGs*	2%	Li G. et al., 2019
Poaceae	BLAST	1E-05	861 conserved Poaceae-specific genes (*CPSGs*)	2%	Campbell et al., 2007
*Aegiceras corniculatum*	BLAST	1E-05	4,823 *Aegiceras*-specific genes (*ASGs*)	12%	Ma et al., 2021
*Citrus sinensis*	BLAST	1E-05	1,039 *OGs* specific to *Citrus sinensis*	4%	Xu et al., 2015
*Citrullus lanatus*	BLAST	1E-05	1,652 *OGs*	7.31%	Ma et al., 2022
*Lagenaria siceraria*	BLAST	1E-05	870 *OGs*	3.87%	Ma et al., 2022
*Sechium edule*	BLAST	1E-05	627 *OGs*	1.63%	Ma et al., 2022
*Cucumis sativus*	BLAST	1E-05	2,524 *OGs*	10.38%	Ma et al., 2022
*Cucumis melo*	BLAST	1E-05	2,287 *OGs*	7.63%	Ma et al., 2022
*Cucurbita moschata*	BLAST	1E-05	2,498 *OGs*	7.76%	Ma et al., 2022
*Trichosanthes anguina*	BLAST	1E-05	529 *OGs*	1.65%	Ma et al., 2022
*Benincasa hispida*	BLAST	1E-05	4,547 *OGs*	16.55%	Ma et al., 2022
*Camellia sinensis*	BLAST	1E-05	1,701 *Camellia*-specific genes (*CSGs*)	3.37%	Zhao and Ma, 2021
*Cajanus cajan*	BLAST	1E-02	266 Phaseoleae-restricted ORFans, 169 out of 266 genes are putative pigeonpea-specific ORFan genes.	0.6%	Varshney et al., 2011

In addition, the *E*-value cutoff is a major contributing factor to the determination of *OGs*. As in the case of the *OGs* discovered in the *Arabidopsis* genome, various numbers of *OGs* were present under distinct *E*-value cutoff (Table 1), with certain parameters (i.e., larger the *E*-value, the lesser are the number of genes obtained and *vice*-*versa*). Similarly, under the *E*-value cutoff of 1E-01 and 1E-04, respectively, 638 *Oryza*-specific genes and 1,926 *OGs* were identified in rice. However, the final numbers of *OGs* were different when the identification methods or programs were modified. Taking into consideration the rice genome, in which 37 *OGs* were obtained under BLAST and BLAT (BLAST-Like Alignment Tool) programs (Jin et al., 2019). Other effective modules or programs include the SMOTE-ENN-XGBoost model (Synthetic Minority Over-sampling TEchnique-Edited Nearest Neighbors-eXtreme Gradient Boosting) (Gao et al., 2020), BIND (BRAK-ER-Inferred Directly), and MIND (MAKER-Inferred Directly) platforms (Li J. et al., 2021), ORFanFinder (Ekstrom and Yin, 2016), combined BLAST and Microarray-based genome hybridization methods (Li G. et al., 2019).

By comparing the available ESTs (Expressed Sequence Tags) and ETs (Expressed Transcripts) from six Solanaceae species on a genomic scale, Solanaceae-specific transcripts in potato, tomato, pepper, tobacco, petunia, and *Nicotiana benthamiana*, were found representing 4,825, 3,151, 1,531, 8,076, 512, and 571 genes, respectively, using an *E*-value cutoff of 1E-10 and the TBLASTX (Rensink et al., 2005). TOGD, a database of *OGs* found in *Triticum aestivum*, was established, and 993 *OGs* were identified as a part of the wheat genome using homology searching against 94 representative plant species, contributing to a complete bioinformatics platform for functional and evolutionary investigations (Gao et al., 2019). Moreover, a comparative database of pangenomes was developed by the name of GreenPhylDB (version 5) for plant genomes that provides orphan gene clusters of 46 species across the plant kingdom using a phylogenetic-based approach, including plants such as *Solanum tuberosum*, *Vitis vinifera*, *Solanum lycopersicum*, and *Helianthus annuus* (Valentin et al., 2021). In addition, ATTED-II is a plant species co-expression database that provides comprehensive information on different co-expression data sets and network analysis tools, as well as a platform for new opportunities to study plant lineage-specific evolution (Aoki et al., 2016).

Furthermore, each gene has been allocated to a phylostratum through the BLAST-based phylostratigraphic approach, which represents the gene’s ancestral evolutionary node (Domazet-Lošo et al., 2007). Following the same approach, the origins of protein-coding genes in *A. thaliana* and rice have been traced, and several young genes have been discovered in both species (Cui et al., 2015). Based on protein sequence clustering, studies comparing non-family (NF) genes in 14 plant species have discovered over 94,000 such genes across these species, which have been classified into five main evolutionary groups (i.e., Viridiplantae wide, angiosperm specific, monocot specific, dicot specific, species-specific), highlighting the evolutionary and functional role of NF genes across species (Ye et al., 2013). However, phylostratigraphy underestimates gene age with a non-negligible probability and the false negative error is prevalent in phylostratigraphy, which can be controlled at least partially by the exclusion of error-prone genes identified *via* realistic simulations (Moyers and Zhang, 2018). Although the percentage of *OGs* in a given species is relatively low, the understanding of *OGs* continues to increase gradually (Wilson et al., 2005).

### Fast Evolving Characteristics of Orphan Genes

On a phylogenetic level, *OGs* have been found to possess a faster rate of evolution in comparison with the evolutionary-conserved genes (*ECGs*). Considering the set of *OGs* present in a given species or lineages tends to share no sequence similarities with any other lineages. Hence, being a signature characteristic of a particular species. Likewise, on a molecular level *OGs* have been shown to possess a shorter gene or protein length, unusual GC content among coding genes (CDS) with a greater number of intron-less genes while a relatively lower percentage of multi-exon genes upon comparison with *ECGs* set (Campbell et al., 2007; Lin et al., 2010, 2013; Varshney et al., 2011; Xu et al., 2015; Plissonneau et al., 2016; Jiang et al., 2018). Furthermore, structural traits related to the process of origin and evolutionary time has revealed that *OGs* have a shorter time of origin than *ECGs*. Younger genes are shorter, higher in the non-synonymous substitution rate (Guo, 2013). Study showed that the elevated ratios of non-synonymous to synonymous SNPs (Single Nucleotide Polymorphisms) in the two sets of *Arabidopsis OGs* are mainly due to the elevated non-synonymous SNP density, indicating that a number of two sets of *OGs* evolve substantially faster than the *ECGs* at the protein sequence level (Lin et al., 2010). For example, due to their short evolutionary time, the genes generated in this time frame have shorter gene lengths (Ma et al., 2021). However, the GC content of CDS, genes, and introns are more variable in diverse species.

According to studies, most variations in GC content, are the consequence of a confluence of factors, including the organism’s living environment and behaviors (Ma et al., 2020). Genetic analysis of *Triticeae*-specific genes (*TSGs*) has shown that nearly one-third of the *OGs* are intron-less and over half of *BSGs* (*Brassica*-specific genes) are intron-less genes, respectively (Jiang et al., 2018; Ma et al., 2020). During evolution, the occurrence of intron-less genes can be associated with lineage-specific or species-specific characteristics within a given species, and *OGs* may be regarded as the prime factors for the existence of biodiversity (Zou et al., 2011). Similarly, ORFans restricted to the Phaseoleae family in pigeon pea tend to possess many of the same properties as ORFans found in other species, such as short length, few introns, and unique GC content (Varshney et al., 2011). Thus, all evidence indicates that *OGs* tend to possess more evolutionarily radical characteristics than *ECGs*. Several studies have revealed that the process of gene origin and extinction occurs dynamically when a given genome gets compromised, leading to the emergence of new genes which get fixated in the genome at various times in evolution (Arendsee et al., 2014; Bhandary et al., 2018). And it is considered nearly impossible to synthesize something as complex as functional protein from scratch (Singh and Wurtele, 2020). Thus, the formation of *OGs* may comprise a vast reservoir of functional proteins with such a tremendous rate of evolution, making them nearly impossible to trace any homology features. Recent study developed a simple method to estimate the probability that a homolog would be detected at a specified evolutionary distance if it was evolving at a constant rate under standard, which indicating that many *OGs* can be explained by homology detection failure (Weisman et al., 2020). And more sensitive synteny-based homology searches successfully find previously undetected homologs for many *OGs*.

## Special Expression Regulatory Mode of Orphan Genes

Expression analysis is a practical and effective method for detecting the *OGs*’ probable function (Xu et al., 2015). The expression patterns of *OGs* are special and widely verified in plants. According to semi-qPCR results and RNA-seq data, *BrOGs* (*B. rapa OGs*) are expressed in diverse organs, tissues, and developmental stages in *Brassica* plants and several *BrOGs* have also demonstrated tissue-specific, organ-specific, or developmental stage-specific expression patterns (Jiang et al., 2018). Similarly, few of the *BrOGs* can be induced by *Plasmodiophora brassicae*, a soil-borne, obligatory, and biotrophic pathogen that infects *Brassica* crops, leading to the development of clubroot and hence reducing overall agricultural output (Chen et al., 2016; Jiang et al., 2018). Thus, the above evidence indicates that *BrOGs* play a certain role in developmental stages, different tissues, organs, and biotic stress responses. *Camellia*-specific genes (*CSGs*) had more tissue-specific expression compared to evolutionary conserved genes (Zhao and Ma, 2021). The *Arabidopsis*-specific genes tend to possess an upregulated expression in mature pollen and under heat stress (Yang et al., 2009). Upon comparison with the evolutionarily conserved set, the *OGs* found in *Arabidopsis* have been more highly methylated in floral tissue (Lin et al., 2010) and have a greater degree of tissue specificity with lower expression levels (Donoghue et al., 2011). *OGs* were transcribed at a lower rate on average.

In *O. sativa*, a larger proportion of these *OGs* have been found to get expressed after sexual maturation and under environmental pressure in comparison to the non-*OGs* (Guo et al., 2007), and have shown relatively high levels of expression in flower tissue, pistil tissue, and root tissue (Yang et al., 2009), while all reported 37 *OGs* have been supported with at least a single evidence of expression (Jin et al., 2019). Similarly, *OGs* have shown relatively high levels of expression in the female flowers, xylem tissue, cambium tissue, and leaf tissue of the *P. trichocarpa* plant (Yang et al., 2009). Other studies have revealed that the detectable expression of *OGs* in young or mature leaves, stem bark, and roots are associated with tissue specific development of that species (Lin et al., 2013).

Likewise, in the case of *V. unguiculata*, *OGs* have also been considered a major contributing factor in maintaining the balance of the agronomic and adaptive traits of domesticated crops in various climatic conditions under artificial selection (Li G. et al., 2019). Lately, it has also been reported that the evolution of nuclear *OGs* can be the result of the involvement of the mitochondrial genome in land plants (O’Conner and Li, 2020). In *C. sinensis*, the *OGs* have been found preferentially expressed in the callus of the plant, while nine of the *OGs* were expressed in response to abiotic treatments (Xu et al., 2015). Similarly, *Aegiceras*-specific genes (*ASGs*) have also been found to get highly expressed on a tissue-specific level whereas 86 *ASGs* co-expressed gene modules are predominantly involved in pathways associated with adversity stress, including plant hormone signal transduction, phenylpropanoid biosynthesis, photosynthesis, peroxisome, and pentose phosphate pathway, as according to weighted gene co-expression network analysis (WGCNA) (Ma et al., 2021). The expression of *OGs* in the case of *A. hypochondriacus* could be a significant element to sustain the extreme tolerance ability to diverse stress conditions among these plants (Cabrales-Orona and Délano-Frier, 2021). Functional annotation of 861 conserved Poaceae-specific genes (*CPSGs*) showed that 346 (40.2%) *CPSGs* are annotated as expressed genes (Campbell et al., 2007).

By this point, our knowledge of *OGs* has been based primarily on comparative genomic studies and expression analysis. However, functions analysis of *OGs* still faces plenty of challenges due to the absence of recognizable domains, functional motifs, and identifiable folds. Fortunately, numerous researchers have made many representative studies while trying to unravel the mystery of the function of *OGs*, which lays a solid foundation for the functional identification of the plant *OGs*.

## Mysterious Functions of Orphan Genes

The vast majority of *OGs* in plants have still not been discovered. Although the function of *OGs* has not been well investigated, *OGs* have been known to play a significant role in the divergence of species (Ma et al., 2021). Analysis of the functionality of *OGs* provides some evidences for its authenticity (Kumar et al., 2015; Jin et al., 2019). To date, researchers have been facing challenges in understanding the encoded functional mechanisms of all the newly discovered *OGs* as well as their source of origination has been a topic of debate (Kell, 1998; Amiri et al., 2003; Rödelsperger et al., 2019; Zhang et al., 2019). Despite being unable to decipher the complete mechanism of action of *OGs*, the studies have provided keen evidence for their major involvement in the processes like primary substance metabolism, response to biotic and abiotic stresses in addition to playing a major role in species-specific traits’ formation (Table 2).

**TABLE 2:** Summary of functional analysis of orphan genes (*OGs*) in plants.

Gene names	Abbreviations	Gene symbols	GenBank accession numbers	Functions	References
*Qua-Quine Starch*	*QQS*	*At3g30720*	EU805808	Carbon and nitrogen allocation across species; genetic and environmental perturbations response; pathogens/pests resistance.	Li et al., 2009; Silveira et al., 2013; Arendsee et al., 2014; Li and Wurtele, 2015; Li et al., 2015; Jones et al., 2016; O’Conner et al., 2018; Qi et al., 2019; Tanvir et al., 2022
*Brassica rapa Orphan Gene 1*	*BrOG1*	*BraA08002322*, *BraSca000221*		Soluble sugar metabolism regulation.	Jiang M. et al., 2020
*Male Sterile 1*	*Ms1*		KX447407, KX447408, KX447409	Male fertility and metagenesis regulation, pollen exine development.	Tucker et al., 2017; Wang et al., 2017
*Male Sterile 2*	*Ms2*		KX533929	Conferment of male sterility.	Ni et al., 2017
*Enhancer of Vascular Wilt Resistance 1*	*AtEWR1*	*At3g13437*	DQ487672	Drought tolerance; fungal pathogens resistance.	Yadeta et al., 2014
*Brassica oleracea Enhancer of Vascular Wilt Resistance 1*	*BoEWR1*			Resistance against fungal pathogens.	Yadeta et al., 2014
*Big Root Biomass*	*BRB*	*SIN_1025576*	MN336257, MN336258, MN336259	Root biomass modulation.	Dossa et al., 2021
*Triticum aestivum Septoria-responsive Taxonomically Restricted Gene 6*	*TaSRTRG6*	*TraesCS1A01G265600, TraesCS1B01G276500, TraesCS1D01G265800*		Septoria tritici blotch resistance.	Brennan et al., 2020
*Triticum aestivum Septoria-responsive Taxonomically Restricted Gene 7*	*TaSRTRG7*	*TraesCS3A01G093900, TraesCS3B01G109200, TraesCS3D01G094200*		Septoria tritici blotch resistance.	Brennan et al., 2020
	*Xa7*		MW467511	Bacterial pathogen resistance.	Wang et al., 2021
*Triticum aestivum Fusarium Resistance Orphan Gene*	*TaFROG*		KR611570	Resistance to the Fusarium head blight disease.	Perochon et al., 2015; Perochon et al., 2019; Jiang C. et al., 2020
*UP12_8740*				Drought resistance.	Li G. et al., 2019
*Oryza sativa ornithine decarboxylase*	*OsODC*	*LOC_Os09g37120*		Biosynthesis of hydroxycinnamoyl putrescine.	Fang et al., 2021
*Oryza sativa putrescine hydroxycinnamoyl acyltransferases 3*	*OsPHT3*	*LOC_Os09g37180*		Biosynthesis of hydroxycinnamoyl putrescine; immunity and cell death regulation.	Fang et al., 2021
*Oryza sativa putrescine hydroxycinnamoyl acyltransferases 4*	*OsPHT4*	*LOC_Os09g37200*		Biosynthesis of hydroxycinnamoyl putrescine; immunity and cell death regulation.	Fang et al., 2021
*Oryza sativa Pyridoxamine 5*′*-phosphate oxidase 3*	*OsPDX3*	*LOC_Os10g23120*		Phenylpropanoid metabolism; bacterial and fungal pathogen resistance.	Shen et al., 2021
*Oryza sativa tyrosine decarboxylase 1*	*OsTyDC1*	*LOC_Os10g23900*		Phenylpropanoid metabolism; bacterial and fungal pathogen resistance.	Shen et al., 2021
*Oryza sativa tyramine N-Hydroxycinnamoyltransferase 1*	*OsTHT1*	*LOC_Os10g23310*		Phenylpropanoid metabolism; bacterial and fungal pathogen resistance.	Shen et al., 2021
*Oryza sativa tyramine N-Hydroxycinnamoyltransferase 2*	*OsTHT2*	*LOC_Os10g23820*		Phenylpropanoid metabolism; bacterial and fungal pathogen resistance.	Shen et al., 2021
*Grain Shape Gene on Chromosome 9*	*GS9*	*LOC_Os09g27590*	MF621928	Rice grain shape and appearance quality regulation.	Zhao et al., 2018
*GRAINS NUMBER 2*	*GN2*			Regulation of grain number, plant height, and heading date.	Chen et al., 2017
*Oryza sativa defense-responsive gene 10*	*OsDR10*		FJ194952	Negative Regulation of pathogen-induced defense response.	Xiao et al., 2009
*Xoo-induced orphan 1*	*Xio1*	*Os09g13440*		Bacterial pathogen resistance.	Moon et al., 2022

### Role of Orphan Genes in Primary Substance Metabolism and Response to Species-Specific Traits

Plant genome sequencing studies with high-quality annotations provide an unprecedented opportunity to investigate the role of *OGs* in plants during environmental adaptation (Ma et al., 2021). The *Arabidopsis QQS* (*Qua-Quine Starch*, *At3g30720*) gene was the first report of *OG* among plants. Li et al. (2009) discovered that *Arabidopsis QQS* has a novel regulatory role. *QQS* has been reported upregulated in *Arabidopsis Atss3* mutants that lack starch synthase III and possess higher leaf starch content. Further experiments have indicated that the accumulation of *QQS* transcript occurs in the response to developmental, environmental, and genetic stimuli.

Similarly, genetic and biochemical evidence has also presented the significant role of *QQS* in the control of starch content (Li et al., 2009). The gene has also been reported to have a significant role in the alteration of plant composition, whereas the gene knockouts tend to develop decreased levels of protein content in leaves and seeds. Despite these molecular modifications, no significant differences in the morphology and development of soybeans expressing *QQS* compared to wild type sibling controls have been identified (Li and Wurtele, 2015). A series of transgenic experiments has shown that the transgenic *QQS* expression increases the protein content of maize and rice, which are highly divergent from *Arabidopsis* (Li et al., 2015). Further research suggests that the QQS protein interacts with the evolutionarily conserved transcription factor AtNF-YC4 (*Arabidopsis* nuclear factor Y, subunit C4, At5g63470), as well as NF-YC4 homologs in maize, rice, and soybean, establishing a role of *QQS* in carbon and nitrogen allocation across species (Li et al., 2015; Jones et al., 2016). AtNF-YC4 is a key element of the NF-Y transcription factor and contains histone-fold domains, which is universal among plant species with a highly conserved peptide sequence (Li et al., 2015; Qi et al., 2019). *AtQQS* and *AtNF-YC4* expression increased total protein content and reduced starch content in both leaf and seed of soybean, rice, and maize, except starch was not detected in the soybean seed (Tanvir et al., 2022). The interaction of an orphan gene from one species with the metabolic network of another species *via* conserved proteins point to novel possible avenues for understanding the complex traits modulation and phenotypic changes in crop species (O’Conner et al., 2018). Furthermore, QQS is proven to be under autonomous epigenetic control and exhibits epigenetic variation among natural accessions, as well as epigenetic variation as well as in wild populations from Central Asia, implying that *de novo* originated genes may be particularly susceptible to epigenetic variation in their early stages of formation (Silveira et al., 2013).

In *B. rapa*, *BrOGs* have been found to produce an extensive impact on soluble sugar metabolism, while *BrOG1* can also probably influence the content of soluble sugar in a SUS-dependent manner (Jiang M. et al., 2020). In this context, a *BrOG* overexpression (*BrOG*OE) mutant library for *A. thaliana* has been developed, with the *BrOG1A*OE mutant producing much higher levels of glucose, fructose, and total sugar content with exceptionally lower levels of sucrose. *BrOG1A*OE mutant has also been shown to possess reduced expression activity of the *Arabidopsis* sucrose synthase (SUS) gene, while the activity of invertase (INV) remained unchanged. The overall decrement in the total sugar content (i.e., fructose, glucose) along with an increase in sucrose contents have been prominent in Chinese cabbage *BrOG1* knockout lines. Similarly, upon comparison with the control plants, the *BOrG1* mutant has a greater activity of BrSUS enzymes with no significant difference in BrINV activity, as well as increased expression of *BrSUS1b*, *BrSUS3*, and *BrSUS5* genes have been reported. This pattern of expression suggests that the phenotype of the *BrOG1* knockout mutants complements that of the *BrOG1A*OE mutants. These findings also indicate that *BrOGs* play a vital role in soluble sugar metabolism. Furthermore, the genomisotopic methodology was developed to give a potent new method for identifying and characterizing orphan biosynthetic gene cluster products (Gross et al., 2007).

To date, the functional characterization of *OGs* is considered a challenging task, although it has been demonstrated that *OGs* may play a role in fundamental agronomic variables (Dossa et al., 2021). Poaceae-specific *Male Sterility 1* (*Ms1*) encoded a phospholipid-binding protein, which was essential for male fertility and metagenesis, and pollen exine development (Tucker et al., 2017; Wang et al., 2017). The grass species-specific *Ms2* (*Male Sterility 2*) genes found dominant in wheat, barley, and *Brachypodium* have been found responsible for conferring male sterility (Ni et al., 2017). This *Ms2* gene appears as an *OG* and is only found in wheat and its close relatives, expression of which has also been reported in anthers of a flower and has been associated with insertion of a retroelement into the promoter. *Ms2* gene also plays a pivotal role in wheat breeding, providing higher yields of hybrid wheat. Similarly, 18 *OGs* were proven to be related to male sterility in watermelon (*Citrullus lanatus*), and the research also confirmed that 182 *OGs* were involved in flower development in cucumber (*Cucumis sativus*), 520 *OGs* may help with the large fruit size in wax gourd (*Benincasa hispida*) (Ma et al., 2022). Study showed that 18 *CSGs* were mainly associated with phenylalanine biosynthesis, biosynthesis of amino acids, pentose phosphate pathway, photosynthesis, and carbon metabolism (Zhao and Ma, 2021). *TSGs* were proven to be associated with reproductive organ development, and 25 flower-specific *TSGs* showed specific expression in developing anthers and had an even higher expression level (Ma et al., 2020). 67 *Conserved Brassicaceae-Specific Genes* (*CBSGs*) was proven to be the pollen determinant, which controlling allele specific pollen rejection in self-incompatible Brassicaceae species (Lin et al., 2010). Overexpression of rice genome unique gene *GN2* (*GRAINS NUMBER 2*) showed less grain number, reduced plant height, and later heading date than control plants, which provided new insight into general phenotypic evolution and more information to elucidate the molecular mechanisms underlying rice grain number (Chen et al., 2017). Higher plants-specific regulator *GS9* (*Grain Shape Gene on Chromosome 9*) was involved in regulating grain shape and appearance quality in rice (Zhao et al., 2018). The interaction of GS9 and OsOFP14 (LOC_Os04g33870) resulting in repression of GS9 transcription activation activity. GS9 together with OsOFP8 (LOC_Os01g64430) and OsOFP14 form a transcriptional complex, which indicating that *GS9* might function in regulating brassinosteroids (BR) pathway, at least partially through its interaction with OsOFP8, thereby modulating rice spikelet development (Zhao et al., 2018).

### Response to Stress

High stress-specificity is regarded as a significant characteristic related to *OGs* (Guo et al., 2007; Bosch et al., 2009; Donoghue et al., 2011). In *B. rapa*, expression patterns of 52 *BrOGs* were assessed upon infection with *P. brassicae* (Jiang et al., 2018). A total of 41 out of 52 *BrOGs* responded to *P. brassicae* treatment, while 39 out of 41 *BrOGs* were found upregulated. This immediate response strongly suggests that *BrOGs* have a significant role in *P. brassicae* stress responses. However, understanding the role of *BrOGs* in *B. rapa*-*P. brassicae* interactions at a molecular level necessitate further research to develop new tools for resistance breeding in Brassicaceae crops and Chinese cabbage in the future. Similarly, In *Arabidopsis*, the Brassicaceae-specific *EWR1* (*Enhancer of Vascular Wilt Resistance 1*) gene and its *Brassica oleracea* (*BoEWR1*) homologs confer resistance to Vascular Wilt pathogens (Yadeta et al., 2014). In *N. benthamiana*, overexpression of *AtEWR1* and *BoEWR1* causes *Verticillium dahliae* resistance. Furthermore, the *Arabidopsis* drought stress tolerance trait has been found augmented by over-expression of *AtEWR1* (Yadeta et al., 2014).

Two *Arabidopsis*-specific POFs (Proteins with obscure features) and a protein with an unknown function from *Arabidopsis* and *Brassica*-specific protein have also been found to elevate oxidative stress tolerance (Luhua et al., 2008). On a similar note, several studies have reported enhanced plant antiviral and antibacterial immune responses upon activation of *AtQQS* and *AtNF-YC4* genes. Likewise, increased resistance/reduced susceptibility toward soybean cyst nematodes, viruses, aphids, fungi, and viruses can also be attributed to the over-expression of these genes in *Arabidopsis* and soybean. Furthermore, by mediating crosstalk between primary metabolism and environmental alterations, the *QQS* gene has also been involved in the optimization and tolerance to biotic/abiotic perturbations (Arendsee et al., 2014; Qi et al., 2019; Tanvir et al., 2022). Recently, 51 *OGs* were proven to be associated with environmental adaptation in watermelon (Ma et al., 2022).

Studies have also described a unique set of *TRGs* present in grain amaranths, a characteristic feature that can be crucial for maintaining a high level of stress tolerance in a variety of conditions (Cabrales-Orona and Délano-Frier, 2021). In parallel, *BRB* (*Big Root Biomass*) is a Lamiales-specific gene, the overexpression of *BRB* in *Arabidopsis* modulates root and shoot traits whereas the same gene has been found responsible to induce drought stress sensitivity, modulating the auxin pathway along with a major impact on root and shoot traits (Dossa et al., 2021). *BRB* is preferentially expressed in sesame root as compared to other organs such seed, leaf, flower and capsule. Similarly, the single motif AACACACAC facilitates the binding of an MYB transcription factor (SiMYB181, SIN_1023179) in the 5′-UTR of *BRB*. The presence of a single motif facilitates the binding of SiMYB181, repressing *BRB* expression while the duplicated motif prevents the binding of SiMYB181, leading to a normal transcription of *BRB* (Dossa et al., 2021).

In the same manner, other TRGs, such as TaSRTRG6 and TaSRTRG7, interact with tiny secreted fungal proteins while promoting resistance toward Septoria tritici blotch (STB) (Brennan et al., 2020). The occurrence of these two genes (i.e., *TaSRTRG6* and *TaSRTRG7*) has been restricted to the Poaceae family, and yeast two hybrid (Y2H) and bimolecular fluorescence complementation (BiFC) assays have revealed that TaSRTRG6 can interact with three different *Zymoseptoria tritici* SSPs (Small secreted proteins), including Zt11, Zt19, and Zt24. Correspondingly, the interaction of TaSRTRG7 has also been observed with two *Z. tritici* SSPs (i.e., Zt16 and Zt18) which suggests the potential role of *TaSRTRG6* and *TaSRTRG7* in STB resistance. Resistance to *Xanthomonas oryzae* pv. *oryzae* infection is conferred by the rice orphan gene *Xa7*, and the Xa7 protein has been discovered to be a necessary factor for resistance response (Wang et al., 2021).

Furthermore, *TaFROG* (*T. aestivum Fusarium Resistance Orphan Gene*) has also been reported to induce leaf resistance in wheat toward *Fusarium graminearum* by interacting with TaSnRK1 (Sucrose Non-Fermenting1-Related Kinase1) (Perochon et al., 2015). TaFROG interacts with TaNACL-D1 (*T. aestivum* NAC-like D1), a wheat transcription factor that provides resistance to the Fusarium head blight (FHB) disease. The co-expression of *TaNACL-D1* and *TaFROG* genes has been reported at the molecular level in response to *F. graminearum*, the pathogenic fungus agent of FHB, and its virulence factor deoxynivalenol (DON) (Perochon et al., 2019). This provides a new source of resistance genes for crop breeding programs. Another study has found that TaFROG modulates host immunity by regulating TaSnRK1 proteasomal degradation (Jiang C. et al., 2020). Further research has revealed that Osp24 (Orphan secreted protein 24) of *F. graminearum* acts as a cytoplasmic effector by competing with TaFROG for TaSnRK1 binding, implying that orphan proteins from both the host and the fungal pathogen play antagonistic roles during their interactions (Jiang C. et al., 2020).

Other *OGs*, like cowpea (*V. unguiculata*) *UP12_8740*, a drought-inducible *OG*, showing the largest expression difference (7.4-fold) in roots under drought stress, tend to confer enhanced drought tolerance in composite transgenic plants (Li G. et al., 2019). The *UP12_8740* gene has been found to exhibit greater expression levels in *UP12_8740*-OE lines than in control lines, indicating that *UP12_8740* is a novel gene providing drought resistance in *V. unguiculata*, according to quantitative RT-PCR analysis. *OGs* can be regarded as a valuable resource for the identification of new genes that are engaged in certain environmental adaptations. A set of *OGs* being part of the Monocot-specific functional hydroxycinnamoyl putrescine (HP) gene cluster (including *OsODC*, *OsPHT3*, and *OsPHT4*) are involved in HP biosynthesis. Further genetic and biochemical analyses of their respective gene cluster have revealed that *OsPHT3* and *OsPHT4* positively regulate rice immunity and cell death in rice (Fang et al., 2021). Rice tribe-specific gene *OsDR10* (*O. sativa defense-responsive gene 10*) functions as a negative regulator in the SA-dependent pathway to balance rice defense response induced by pathogen infection (Xiao et al., 2009). A defense-related *Oryza*-specific orphan gene *Xio1* (*Xoo-induced orphan 1*) specifically induced by the bacterial pathogen *X. oryzae* pv. *oryzae* (*Xoo*) in an immune receptor XA21-dependent manner (Moon et al., 2022). Overexpression of *Xio1* showed enhanced resistance to *Xoo* and reactive oxygen species accumulation.

Similarly, transposon element (TE)-assisted formation of hydroxycinnamoyl tyramine (HT) gene cluster (including *OsPDX3*, *OsTyDC1*, *OsTHT1*, and *OsTHT2*) is specific to *Oryza* AA genome lineage, which contributes to enhanced disease resistance and often exhibits co-expression in a specific tissue or under certain stresses (Shen et al., 2021). In a conducted study, 1,007 genes of unknown functions were taken from *Arabidopsis* from which the ones containing *OGs* were selected and assessed for the response of their corresponding homozygous T-DNA insertional mutants to hypoxia, cold, heat, salinity, oxidative, and osmotic stresses (Luhua et al., 2013). Out of which 832 out of 1,007 mutants displayed tolerance or sensitivity to multiple abiotic stress treatments, implying that genes with unknown functions may play a role in abiotic stress response signaling or general acclimation mechanisms. Study showed that nearly one third of the *TSGs* were stress-responsive and inducible under abiotic and/or biotic stresses (Ma et al., 2020). These findings provide a concrete library of genes that can play a significant role in plant defensive strategies against various kinds of environmental conditions.

Although the functions of *OGs* are still difficult to predict by homologous comparison or other bioinformatics approaches, recent studies indicated that *OGs* implicated in various abiotic and biotic stresses response, plant growth and development regulation, primary substance metabolism, and species-specific adaptation. Functional analysis of these *OGs* indicated that they are of great importance for plant improvement by using mutants or altering their expression, and the significance of *OGs* characterization is just showing up. However, an enormous amount of work is needed to unravel the mysterious functions of *OGs*. *OGs* are prevalent in life forms and are important for the genome evolution and phenotypic changes (Ma et al., 2021). The generation of *OGs* can contribute to the morphological diversity of distinct species, and many *OGs* may be very good models for studying of the evolution of organisms.

## Concluding Remarks and Future Perspectives

**FIGURE 1:**
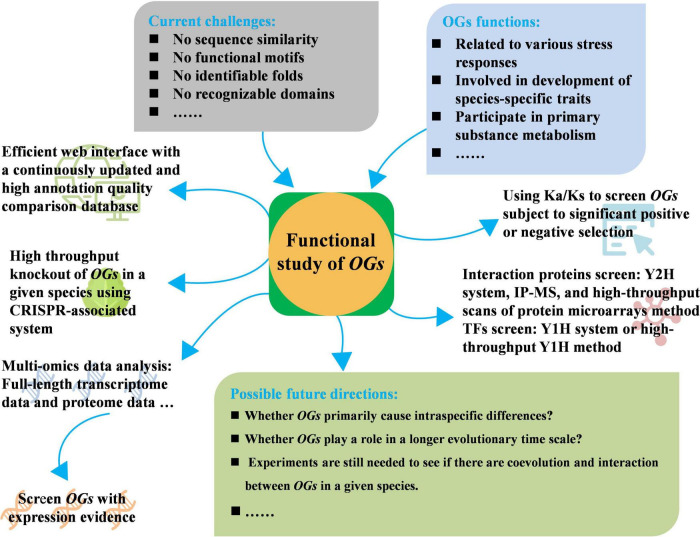
Current challenges, adoptable strategies, and possible future directions of functional study of orphan genes (*OGs*).

Orphan genes are defined as the genes with no observable homolog in other lineages, and hence are a particular characteristic of an individual species. The availability of a large collection of genomic databases has made it possible to identify and characterize the functional characteristics of *OGs*. The present study has revealed that the *OGs* play a specific role in primary substance metabolism, species-specific traits’ formation, and response to diverse stresses, making them important for lineage-specific adaptations (Figure 1). *OGs* have a shorter origination time and distinctive features relative to *ECGs* among all species (Ma et al., 2022). Gene structure analysis showed that OGs exhibit significantly shorter protein length, which mainly attributed to the fewer number of exons, and younger genes have fewer exons. *OGs* possess unusual GC content among CDS compared to *ECGs* set. Although *OGs* in different plants have no sequence similarities, they share similar genic features, implying their evolutionary mechanisms could be similar (Ma et al., 2020). The evolutionary origin of *OGs* is a microevolutionary process, and the study of how *OGs* arise and differentiate is essential to explain the generation and succession of novel phenotypes and eventually biodiversity (Ma et al., 2022). The relevance between the adaptive evolution and generation of new agronomic traits still needed to be determined. For example, the divergence of *B. rapa* from *A. thaliana* was under a relatively short evolutionary time about 12.4–13.4 million years (Yang et al., 2006; Liu et al., 2014), but some *B. rapa* plants displayed extreme morphological characteristics as a result of artificial selection during domestication and breeding, such as leafy heads of Chinese cabbage (Cheng et al., 2016), which is the completely different phenotype from *Arabidopsis*. Many *OGs* may appear and become fixed and functional in the *B. rapa* genome during this relatively short evolutionary time, therefore, the relationship between these *OGs* and leafy head formation of Chinese cabbage will become a very interesting research direction. Furthermore, Chinese cabbage is a long-day biennial plant and needs a vernalizing stimulus and subsequent long-day conditions for bolting (Song et al., 2015; Su et al., 2018). However, *A. thaliana* is a facultative long-day species and flowers most quickly in the long photoperiods during spring and early summer, and can display either a summer- or winter-annual flowering phenotype (Kinmonth-Schultz et al., 2021). Thus, whether these *OGs* are involved in the different flowering habits control is also the focus of future research, which may provide insights and gene resources into bolting resistance regulation, especially in solving the problem of premature bolting of spring Chinese cabbage cultivation.

The current study also highlights the interactions made by *OGs* on a molecular level with more conserved cellular components, making them function across species, such as in the case of QQS. Thus, the predominant molecular genetic mechanisms controlling *OGs* emergence, turnover, and fixation remain to be elucidated. Although the mystery of *OGs* origins is gradually being unraveled, how some *OGs* can play a vital role in a very short evolutionary time and their specific functions need to be further studied, still, the mechanism of action and functional characteristics of *OGs* require further research in this arena. For *OGs*, we still need to continue to explore and discover, but we have now begun to trace the traces of their ancestors, and it seems that we can’t find the families of most *OGs* because they have no families. What is the relationship between the gene structures of *OGs* and its origin mechanism, origin motivation, and the influence of gene structure on biological function and phenotype? How does an *OG* participate in the complex biological network of an organism and perform biological functions? What drives the creation and retention of *OG*? These issues need to be further studied. At present, the research on the function of plant *OGs* started late, but the function of *OGs* can’t be underestimated. *OGs* are important for viability to the plants, and the discovery of many protein-protein interaction models has gradually uncovered the mysterious functions of *OGs*, such as the interaction of AtQQS and AtNF-YC4. *OGs* were not merely results of random evolutionary development, they may indeed be an essential part of organisms’ key trades (Tanvir et al., 2022), and the mysterious function of *OGs* still needs comprehensive exploration.

Although some progress has been made in the study of *OGs*, the research scope remains limited to a few species, the breadth of *OGs* function needs to be further explored, and some adoptable strategies may play a role in the study of the function of *OGs*. First, annotation quality of the database and search algorithms have a significant impact on the identification of *OGs*, one of the problems to be solved is to create an efficient web interface with a continuously updated and high annotation quality comparison database. Although comparative pangenomic database GreenPhylDB provided sequence information and protein domain signatures of *OGs* for several plant species, multiple expression data of *OGs* were not integrated, such as the transcriptome sequencing data under different conditions. Therefore, it is necessary to establish a new and comprehensive database for plant orphan gene comparison and annotation. Second, how do *OGs* participate in the complex biological network of organisms and play biological roles, as well as how do they participate in the life activities of important tissues of organisms, high-throughput knockout of *OGs* in a given species using CRISPR (Clustered Regularly Interspaced Short Palindromic Repeats) may significantly speed up the characterization of *OGs* function, clarifying the biological functions of *OGs* and their influence on phenotypes. CRISPR-associated system is a powerful genome-editing tool, combined with the development of next-generation sequencing and many other high-throughput techniques, which have thus been quickly developed into a high-throughput engineering strategy in plants (Huang et al., 2022). CRISPR-associated system has resulted in the development of powerful new screens to test gene functions at the genomic scale (Gaillochet et al., 2021). Mediated mutagenesis of CRISPR-associated system has increasingly been used to reveal or validate functions of candidate genes identified in genetic studies of various plant species (Puchta et al., 2022). Large-scale mutation have been mediated by using CRISPR-associated systems, which have been performed in tomato (Jacobs et al., 2017), rice (Meng et al., 2017; Ma et al., 2019), soya bean (Bai et al., 2020), maize (Liu et al., 2020), and cotton (Ramadan et al., 2021), which will provide theoretical basis and practical guidance for the functional study of *OGs*.

Due to the large number and proportion of *OGs*, it is very important to select suitable candidate *OGs* for their functional verification. Third, the use of multi-omics data, such as using full-length transcriptomes data to screen *OGs* with direct transcription evidence or using proteome data to screen *OGs* with expression evidence for subsequent verification. Multi-omics data will enable yet more powerful analyses and provide a powerful high-throughput approach to place them in a physiological context, and *OGs* are a wildly deviant group often with a highly variable pattern of expression, thus, even more than for ancient genes, it is important to use a wide variety of experimental conditions in any meta-analysis to evaluate *OGs* function (Bhandary et al., 2018). Recent study showed that 23 Holm oak (*Quercus ilex*) unique enzymes were related to the biosynthesis of hormones and secondary metabolites, which were identified through proteome analysis (López-Hidalgo et al., 2018). Fourth, using the genomic data of related species, Ka/Ks (the ratio of non-synonymous substitution and synonymous substitution) is used to determine whether there is selection pressure on candidate *OGs*, and the genes subject to significant selection may have important functions. Thus, the significance of choosing such *OGs* to study their functions may be greater. Recently, about 90% of the Carboxylesterase (*CXE*) genes of the four cotton species were found to have a Ka/Ks ratio of less than 1 after determination, which indicating that the family genes were affected strong purification options (Rui et al., 2022). The *Brassica napus BnSDGs* (SET domain genes) were proven to be under strong purifying selection during the evolution after Ka/Ks determination (Sehrish et al., 2022). Moreover, the interaction proteins of OGs of interest can be screened by different methods, such as Y2H system (Fields and Song, 1989), IP-MS (Immunoprecipitation-Mass Spectrometry) (Marcon et al., 2015), and high-throughput scans of protein microarrays method (Zhu et al., 2001; Struk et al., 2019). The functions of these OGs can be studied from the known functional interaction proteins. The construction of transcription factors (TFs) cDNA library can screen the potential interaction TFs of *OGs* with specific nuclear localization and no transcriptional activity by the yeast one-hybrid (Y1H) system, and clarify the function of target genes by analyzing the function of interaction TFs. Y1H system has become an important technique for detecting physical interactions between sequence-specific regulatory TFs and their DNA target sites (Reece-Hoyes and Marian Walhout, 2012). Recently, high-throughput Y1H screen has developed to provide a simple and effective strategy to identify TF-promoter interactions using a DNA fragment as bait (Li Z. et al., 2019), which will accelerate the identification of TFs that regulate the expression of *OGs*.

Although the evolutionary examples of *OGs* are described to determine whether they are preferentially included in the specific nodes of the regulatory network, future research is needed to determine whether they primarily cause intraspecific differences or play a role in a longer evolutionary time scale. Furthermore, experiments are still needed to see if there is coevolution and interaction between *OGs* in a given species. In brief, *OGs* have the potential to be utilized in the improvement of important agronomic traits and broaden gene resources for plant breeding.

## Author Contributions

MJ and HL conceived the original idea. All authors contributed equally to writing and final editing, contributed to the article, and approved the submitted version.

## Conflict of Interest

The authors declare that the research was conducted in the absence of any commercial or financial relationships that could be construed as a potential conflict of interest.

## Publisher’s Note

All claims expressed in this article are solely those of the authors and do not necessarily represent those of their affiliated organizations, or those of the publisher, the editors and the reviewers. Any product that may be evaluated in this article, or claim that may be made by its manufacturer, is not guaranteed or endorsed by the publisher.
